# Supplementary material for: Oral and rectal colonization of Gram-negative antimicrobial-resistant bacteria and Methicillin-resistant Staphylococcus aureus in one long-term care facility and changes in professional oral hygiene care: Cross-sectional and interventional study
Source: PLoS One. 2026 Mar 3;21(3):e0343092. doi: 10.1371/journal.pone.0343092 (PMC12956076; doi:10.1371/journal.pone.0343092)
Supplement: Attachment — Submitted filename: Response to Reviewers.docx [file pone.0343092.s005.docx]

Dear Editors and Reviewers

Thank you very much for reviewing our manuscript and offering valuable advice.

We have addressed your comments with point-by-point responses, and revised the manuscript accordingly.

**Response to reviewers’ comments:**

Reviewer #1 (Review Comments to the Author):

Thank you very much for your kind suggestion.

1. *Check the accuracy of the title.*

Ans: According to the suggestions, we revised the title (Line1-3).

2. *Table 2 and Table 3, detection of CTX-M genes are group specific or specific CTX-M types?*

*Detection by PCR might be CTX-M- group specific, so that CTX-M1, CTX-M2, and CTX-M9 should be rephrased as CTX-M-group-1, CTX-M-group-2, and CTX-M-group-9 respectively.*

*Without sequencing of CTX-M gene covering the whole ORF, the specific CTX-M- type could not be assigned.*

Ans: According to the suggestions, we rephrased them (Line 162-163, 288, Table 2 and Table 3).

3. *Table S1, references of primers are not listed in reference section. These references should be described in foot note of Table S1.*

Ans: According to the suggestions, we described the references in foot note of Table S1.

Reviewer #2 (Review Comments to the Author):

Thank you very much for your helpful recommendation.

1- *In this manuscript, the pronoun “we” has been used frequently (67 times). In scientific writing, it is preferable to avoid personal pronouns. Please replace them with formal expressions such as “this study,” “the present study,” or “the current study.”*

Ans: According to the suggestions, we revised them except where necessary (3 times, Line 395, 488, 490).

2- *Please add a clear Conclusion section following the Discussion section.*

Ans: According to the suggestions, we added the Conclusion section (Line 482-485).

3- *It is recommended to include a list of abbreviations at the end of the manuscript for better readability.*

Ans: According to the suggestions, we added the list of abbreviations (Line 606-621).

4- *Several references are outdated. Kindly update the reference list by citing more recent studies published from 2018 onward.*

Ans: According to the suggestions, we revised them.
